# Exploring the “how” in research partnerships with young partners by experience: lessons learned in six projects from Canada, the Netherlands, and the United Kingdom

**DOI:** 10.1186/s40900-022-00400-7

**Published:** 2022-11-17

**Authors:** Linda Nguyen, Bente van Oort, Hanae Davis, Eline van der Meulen, Claire Dawe-McCord, Anita Franklin, Jan Willem Gorter, Christopher Morris, Marjolijn Ketelaar

**Affiliations:** 1 School of Rehabilitation Science, McMaster University, Hamilton, ON Canada; 2 CanChild Centre for Childhood Disability Research, McMaster University, Hamilton, ON Canada; 3 Sibling Youth Advisory Council, Hamilton, ON Canada; 4 The Netherlands Organisation for Health Research and Development, The Hague, The Netherlands; 5 Supervisory board of Stichting JongPIT, Amsterdam, The Netherlands; 6 Cumming School of Medicine, University of Calgary, Calgary, AB Canada; 7 Bachelor of Health Sciences Program, McMaster University, Hamilton, ON Canada; 8 School of Education and Sociology, University of Portsmouth, Portsmouth, UK; 9 UMC Utrecht Brain Center, University Medical Center Utrecht, Utrecht, The Netherlands; 10 Department of Pediatrics, McMaster University, Hamilton, ON Canada; 11 PenCRU (Peninsula Childhood Disability Research Unit), University of Exeter Medical School, University of Exeter, Exeter, UK; 12 De Hoogstraat Rehabilitation, Utrecht, The Netherlands

**Keywords:** Involvement, Partnership, Disability research, Young people, Adolescents and young adults, Participatory research, Lived experience, Decision-making

## Abstract

**Background:**

Involvement of young partners by experience in research is on the rise and becoming expected practice. However, literature on how to promote equitable and meaningful involvement of young people is scarce. The purpose of this paper is to describe and reflect on different approaches between researchers and young partners by experience based on six research projects conducted in Canada, Netherlands, and United Kingdom.

**Methods:**

From six exemplar research projects, at least one researcher and one young partner by experience were asked to collaboratively (1) describe the project; (2) summarise the values and practicalities of the project; and (3) reflect on their partnership. Thematic analysis was applied to the findings from these reflective exercises, which included meeting summaries, recordings, and notes.

**Results:**

All projects shared similar values, including mutual respect between all team members. Young partners were offered a variety of opportunities and approaches to being involved, for example in recruiting participants, co-analysing or (co-)presenting results. Supports were provided to the teams in a variety of ways, including organizing accessible meetings and having dedicated facilitators. Regular and proactive communication was encouraged by using asynchronous modes of communication, establishing reference documents, and a personal approach by facilitators. Facilitators aimed to tailor the needs of all team members by continuously discussing their preferred roles in the project. While most projects did not offer formal research training, various learning and skill development opportunities were provided throughout, including presenting skills or advocacy training.

**Conclusion:**

With this paper, we demonstrated the value of reflection, and we invite others to reflect on their partnerships and share their lessons learned. Our recommendations for involvement of young people in research are: (1) Remember that it is okay to not know what the partnership might look like and there is no single recipe of how to partner; (2) Take the time to invest in partnerships; (3) Provide ongoing opportunities to reflect on partnerships; (4) Consider how to balance the power dynamics; and (5) Consider how to incorporate diversity in the background of young partners in research.

**Supplementary Information:**

The online version contains supplementary material available at 10.1186/s40900-022-00400-7.

## Plain English summary

In more and more projects, researchers and young people are working together in partnership; but there is little guidance about *how* to organize this partnership. In this paper, we share what partnerships in six projects from Canada, Netherlands, and United Kingdom looked like, so that others can be inspired. To do so, a researcher and a young partner from each project were asked to together: (1) describe their project, (2) summarize the practical details about the collaboration and (3) think about things that went well or could be improved. We found that all projects had the same beliefs important to partnerships, like having respect for each other. Young people could work on parts of the project they liked in a way that worked for them. They were supported by staff, could join meetings and were appreciated for their work. Clear communication during and in-between meetings was helpful. Youth were often asked about the role they wanted in the project. While there was often no formal training on how to do research, there were many opportunities to learn. We offer six recommendations to researchers and young people who want to partner together: (1) It is okay to not know what the partnership will look like and there is no single recipe of how to partner; (2) Take your time; (3) Discuss how the partnership is going; (4) Think about who is doing what and why; (5) Consider the diversity of young partners. We hope others will share their experiences.

**Supplementary Information:**

The online version contains supplementary material available at 10.1186/s40900-022-00400-7.

## Introduction

In recent years, partnerships between researchers and young people throughout the research project is increasing and becoming an expected practice. Young people are often experts by experience, which can refer to any adolescent or young adult with lived health or other experience in the context under study. This paper focuses on the involvement in research projects of young people who have a disability or a chronic health condition or are a family member of an individual with a chronic condition (e.g., siblings). Increasingly, researchers have responded to the right of young people by experience to express their views freely in matters that concern them [1], by involving them as partners in health-related research and directly consulting for their ideas and opinions [2–5]. Despite the imperative value and right to involve young people in research, there is limited information in the literature about *how* to enhance and therefore ensure their equitable involvement.

There are many terms to describe the involvement of experts by experiences that are often used interchangeably, including patient and public involvement, patient engagement, engagement, authentic stakeholder engagement, involvement and participation [6]. In this paper, we refer to the definition by the National Institute for Health Research in the United Kingdom, which defines involvement as a situation in which members of the public are actively involved in research studies (as co-applicants, advisory or steering members, joint grant holders) and inform research priorities as well as research development and conduct [7].

The involvement of young people in research can bring mutual benefits to young people, the research itself, and to researchers [8, 9]. Young people involved in research projects may gain new knowledge and skills [10–13]. They may also have opportunities to develop or broaden a social network [10, 13] and increase their independence, confidence and self-esteem [10, 11, 14]. Positive impact on the research itself has been reported too, including enhanced relevance of the research, access to hard-to-reach youth, and further application of the research [15, 16]. The results of the research projects may produce outcomes enhanced by partnership, such as more user-friendly products and/or stronger calls to action and advocacy for future research [16]. Researchers have also reported personal benefits in partnering with young people, including increased motivation, satisfaction, and understanding about the perspectives of young people and end-users to enhance data collection and data analysis methods [10, 17, 18].

Given the value of engaging young people in research, it is important to think about the various aspects and stages of research, and the way how young people may want to be involved. A scoping review by Van Schelven and colleagues [19] reported on the varying levels of involvement of young people with a chronic condition in health research projects [19]. The scoping review identified that the involvement of young people is a continuum of activities, and the ways that young people can influence the research project can range from being informed to being a decision maker [19]. The included studies in the scoping review have involved young people in *one* part of the research project [11, 12, 20, 21], but there is increasing consensus that there should be opportunities to involve young people throughout the entire research process, such as developing and prioritizing research questions, contributing to the study design, increasing accessibility of recruitment and advertisements, offering unique perspectives for data analysis, and improving the use and applicability of the research findings [7, 10, 15, 16, 20, 22, 23].

Many publications have reported on the value of “why” we involve young people in research and “what” activities they have been involved in. However, there continues to be an underreporting about *how* to involve young people in various stages of research [6, 19, 24]. The literature currently offers insufficient details about the context and mechanisms of how specific strategies were used to organize partnership with young people in research [24]. Reflections from research teams who engage with experts by experience can be beneficial to share with others who wish to form similar partnerships in research. In work conducted by Liabo et al. [25], researchers and public advisors from three involvement groups comprised of adults and parents participated in a reflective exercise in which they were asked about their perspectives about good practices for involvement identified from the literature, as well as challenges and what works well for their communities. Good practices for involvement from the literature were summarized as a framework, including an iterative process of evaluation of the values and practicalities for involvement [25]. The reflective framework proposed by Liabo and colleagues enables teams to reflect on their values, as well as the practicalities of how to achieve these values [25].

The purpose of this paper is to illustrate and reflect on different approaches to engage young people in research based on various research projects conducted in Canada, the Netherlands, and the United Kingdom, including the values, practicalities, and involvement in research, to share ‘how’ young people have been and can be involved as partners in research.

## Methods

There were six different projects that we reflected on as individual case studies; these were two projects each in Canada, the Netherlands, and the United Kingdom.^1^ Some members of the team (LN, BvO, and MK) had conversations about how partnerships with young people were similar and/or different based on their experiences in partnering with young people and researchers in projects. Based on these conversations, these members discussed about the importance of sharing these reflections as a paper. During the initial stages of conceptualizing this paper, they reached out to invite other members who may be interested in partnering in this paper. Young partners from each of these projects were invited to be involved in different roles (for example, as a listener, co-thinker, advisor, or partner as described in the Involvement Matrix [26]). An invitation with a guiding Terms of Reference was sent in the native languages of English or Dutch to young people about what their involvement might look like for this paper. For young partners who expressed an interest in being involved in this paper, they were invited to attend the team meetings based on their interest and role that they would like to have. Team meetings with both researchers and young partners were often held on a bimonthly basis since October 2020 to January 2022 to discuss how to share our reflections and lessons learned in this paper. Multiple strategies were used to establish a welcoming and inclusive environment, including sending a poll to ask for the availability of team members, sending meeting agendas, having a brief check-in with every member at the beginning of each meeting, recording the team meetings, and sharing a written summary after each meeting.

We use the term “project team” to refer to the case study project team and “author team” to describe our full team of co-authors. All young people in each project team are referred to as “young partners” in which they have emphasized the importance of highlighting the expertise of their lived experiences. The GRIPP2 short form is an international checklist that was created to enhance the quality, transparency, and consistency about patient and public involvement in research [27], and this checklist was used as a guideline to report on the involvement of young people in work described in this paper.

Each project team were asked to complete the following reflective exercises as dialogues:

1. Describe the project, including its aims, location, duration, number of young partners, roles and activities;
2. Summarise the values and practicalities of the project informed by the framework of involvement from Liabo and colleagues [25];
3. Reflect on the partnership between young partners and researchers using guiding questions (adapted from Liabo and colleagues [25] including:
  - What made/makes the group work?
  - What would we have done differently (and if applicable, moving forward in ongoing projects)?
  - Recommendations and key messages to others engaging with young partners in research?

The reflections also focused on how the practicalities reinforced the values from all project teams.

To complete these reflective exercises, each team met separately to have a conversation and write a document of key points of values and practicalities that they would like to share. The values are defined as ideals of importance that were held by project teams and included:

- inclusivity to provide equal opportunities for young people to be involved;
- partnership in which researchers and young people showed respect for each other’s contributions;
- purposeful involvement about why young people were involved in the projects;
- transparency with open and honest communication;
- and value of different kinds of knowledge [25, pp. 5].

The practicalities, defined as something that enables the values, included:

- support to young partners;
- capacity building in which there was co-learning between young partners and researchers with training for both groups;
- proportional involvement that was tailored to the needs of the research and young partners, with pragmatic decisions being made;
- communication that needs to be proactive;
- and involvement throughout the research by young partners [25, pp. 5].

The information from each reflective exercise was presented at the author team meetings, with discussions about similarities and differences across case study projects.

During the process of co-writing this paper, the full author team meeting was held with an introduction about writing guidelines such as the steps involved with writing a scientific paper, author roles, expectations, and timelines [28–30]. Thematic analysis was applied to the documents from these reflective exercises, which included meeting summaries, recordings, and notes from each project team. The first author (LN) used principles from deductive thematic analysis using the framework of values and practicalities outlined by Liabo and colleagues [25], as well as inductive thematic analysis to identify new information that did not fit in these categories of values and practicalities [31]. Information from this analysis was summarized to describe the similarities and differences across project teams about the values, practicalities, and reflections of the partnerships between researchers and young partners. A first draft of the following documents were shared: tables of values and practicalities based on thematic analysis across all project teams, a summary of similarities and differences across all project teams, and a manuscript. Each project team continued to meet separately to share feedback for these documents, and author team meetings provided an opportunity to discuss the key elements that should be included in the paper such as key similarities and differences across project teams, as well as key messages to share from our overall partnerships between young partners and researchers.

## Results

Across all case study projects, young partners ranged in age from 12 to 38 years old who shared their lived experiences during adolescence and young adulthood to inform the study project. Young partners had lived experiences, in which they either had a chronic health condition or disability, or had a sibling with a chronic health condition or disability. A brief description of each project is provided in Table 1. The details of the background of our young partners and the projects are provided in Additional file 1. A summary of values is presented, which were similar across all projects. We highlight the practicalities as the focus of this paper to illustrate *how* young partners have been involved in each project.

**Table 1:** Project descriptions

Case study project	Description
Care and future prospects (CFP)^a^	The Youth Panel CFP was a panel of young partners by experience, founded to advise the CFP program in the Netherlands on which projects to subsidise to help young people with a chronic health condition. The CFP panel continued to expand its work and influence to improve the social position of young people with chronic health disorders in five areas: care, school, work, sport and empowerment. Today, the panel merged into a foundation, JongPIT, which is completely managed by (young) experts by experience.
Participation in Perspective (PiP) Project	Youth with lived experience of cerebral palsy, known as Ambassadors, were involved in various stages of the PiP research project that aimed to understand the experiences and stories of adolescents with cerebral palsy about participation and autonomy in the context of school, work, sports and care.
BrothErs and Sisters involvement in health care TranSition for youth with Brain-based disabilities (BEST SIBS) Study	Young adult siblings as members of the Sibling Youth Advisory Council (SibYAC) have been involved in the design and execution phase of a qualitative BEST SIBS Study, which aims to understand the roles and responsibilities of siblings and have a brother or sister with a neurodisability.
READiness in Youth fOR transition Out of pediatric Care Brain-Based Disabilities (READYorNot™ BBD) Project	The Patient and Family Advisory Council (PFAC), comprised of both youth and parents, was developed to partner in the READYorNot™ BBD Project, a patient-oriented research project. The project aims to develop and evaluate the effectiveness of the MyREADY Transition™ BBD App to empower youth during their transition from pediatric to adult health care.
Voice, Inclusion, Participation, Empowerment, Research (VIPERS) Project^b^	Disabled youth, known as Vipers, co-led and delivered the VIPER Research Project that aimed to examine the participation of disabled children and young people in decision-making at a strategic level within services across England.
Research into Practice: Skilled Team with Ambition, Rights and Strength (RIP:STARS) Project	The RIP:STARS Project is a disabled young people co-led research collective which undertakes research studies to inform policy and practice concerning the rights of disabled children and young people both nationally and internationally. Young people involved in this project referred to themselves as the RIP:STARS.

^a^The CFP Panel have merged in 2020 with the Ervaringskenniscentrum Jong & Perspectief and All of Me in JongPIT. JongPIT’s mission is to make it possible for all young people (15–30 years old) in the Netherlands with a chronic condition to fully participate in society.

^b^While the VIPERS Project has been completed, young partners from the VIPERS Project developed the idea and concept for the research that has been carried forward with the RIP:STARS Project.

## Summary of values across all projects

The values identified from the reflective exercises are summarized across all projects. There were equitable opportunities with considerations about how young people could be involved, while recognizing that they might also need to balance their commitments with their health, home, school, and work. Young partners were welcome to join the projects at any point, and in all projects, they could take a step back from the projects and rejoin when they are available. The partnership was built on a foundation of respect for each other’s contributions and roles to work together as a team. In Canada, and specific to the partnerships with young partners in the BEST SIBS Study and READYorNot™ BBD Project, there was a commitment to the partnership between young partners by experience and researchers, who may have already been familiar and been involved with other research projects at the CanChild Centre for Childhood Disability Research [32] and wanted to continue to build on patient-oriented research projects. Furthermore, there was a vision to enhance the work of patient-oriented research work from the Strategy of Patient-oriented Research (SPOR) from the Canadian Institutes of Health Research (CIHR) and the CIHR-SPOR Patient-Oriented Research Fellowship Award that was funding the READYorNot™ BBD Project, as well as the doctoral studies and the BEST SIBS Study, respectively [33].

In all projects, the contributions from young partners were appreciated which were shown through verbal comments, financial compensation, opportunities to contribute to all aspects of the project, and group bonding activities. There was a purpose for inviting young people to be involved in different aspects of the project, which was communicated from the beginning. All projects valued the different kinds of knowledge being shared by young partners and researchers. The perspectives and expertise shared by young partners were encouraged and taken into consideration in each project. In addition, all projects had the value of having open and honest communication between young partners and researchers to provide clarity about each step of the project. Accessible methods were adopted to ensure that young partners were supported in their involvement. For example, in-person meetings for the CFP Panel were held in a wheelchair accessible building. In the BEST SIBS Study and PiP Project, dietary restrictions were taken into consideration when food was provided. Flexible formats of communication were used based on the preferences of young partners in all projects, such as email, Facebook, WhatsApp, and Zoom. Documents were written in plain language with large text, where possible. However, the role of young partners could sometimes be unclear with a lack of transparency about how decisions were being made. Project teams reflected about how to improve communication methods and ensure transparency in decisions being made. Collective reflections by the project teams also suggested the need for more time and space to reflect on the changing involvement of youth throughout the project as well as internal changes in the project for a variety of reasons, such as practical changes. These reflections could help to evaluate the partnership throughout the project and identify good practices moving forward as a team. Additional file 2 provides further information about our values.

## Practicalities and reflections

### Involvement throughout the research

Young partners by experience were asked about the different activities that they would like to be involved in. Additional file 3 outlines the involvement of young partners as they reflect about their experiences in partnering in research projects. The activities that young partners have been involved with provides context about the partnership between young partners and researchers in each project. We then further describe our reflections on the practicalities outlined in the proposed framework by Liabo et al. [25] to describe *how* young partners and researchers partnered together in each project team. Additional file 4 provides details about our reflections on the practicalities and what we could have done differently in our partnerships.

#### Preparation phase

During the preparation phase of a research project, young people were involved in a variety of activities which included reviewing plain language summaries of grant applications by the CFP Youth Panel and the SibYAC with the BEST SIBS Project. Most projects were focused on the research activities to start conducting a project, which included the development of the research question, providing feedback on questionnaires, identifying study methods, and developing the interview guide and recruitment materials. The PiP Project highlighted how it was important to involve young partners to draft the recruitment letter for participants in the research, in which young partners ensured that the language was appropriate, and the letter was appealing to other young people to participate in the study. Similarly, the recruitment materials were co-created in the RIP:STARS and VIPER Projects and BEST SIBS Study. Some young partners contributed to the co-development of the recruitment videos. For example, young partners in the READYorNot™ BBD Project drafted, scripted, and provided testimonial videos for the recruitment videos [34]. They further refined and launched the recruitment strategy on social media, such as through their personal networks on Facebook and Twitter. Similarly, young partners in the BEST SIBS Study provided testimonials about the importance of the study to encourage individuals to participate in the recruitment video [35], which they shared in their personal social media networks. In addition to participant recruitment, young partners were also involved with the design of the study; in the BEST SIBS Study, VIPER, RIP:STARS and PiP Project, young partners and researchers further discussed study methods that would be novel and engage with young people as participants in the study, such as with photo elicitation in which participants could share photographs and describe stories during the interviews [36]. For young partners of the READYorNot™ BBD Project, they were involved with the co-development of an App and prepared the e-learning modules to train research assistants who were conducting the study. In the RIP:STARS and VIPER Projects as the disabled young people were to undertake the whole research project themselves, preparation involved training in research methods and ethics. These examples of activities demonstrate how young people were involved in a variety of ways as part of the team to prepare the study.

#### Execution phase

During the execution of the study, all projects considered how to involve young people in all activities that they might express an interest. Young partners in the VIPERS and RIP:STARS Projects undertook all aspects of the research projects, including designing their sample, gathering data via interviewing participants and facilitating workshops with other disabled children, co-developing the analysis framework and conducting data analysis, co-writing the final report, developing policy and practice recommendations and implementing the evaluation of the project. As co-leaders of the research, they were trained and supported by academics to guide them in producing rigorous research, but final decisions and the execution of the study was delivered by the young people. Similarly, there was an opportunity for young partners in the PiP Project to be involved in the analyses and interpretation of the interviews. Young partners in the BEST SIBS Study piloted the interview guide and had the opportunity to be involved with the analyses of the interviews. A graduate trainee and first author of this paper (LN) learned alongside with the partners in the BEST SIBS Study about how to provide training and involve young partners in the BEST SIBS Study throughout the process of data analysis. LN developed a brief 10-minute tutorial to explain qualitative terms to young partners. There was an iterative process with multiple discussions among the team to understand and interpret the data. The discussions took place in approximately 1-hour meetings. In one discussion, a senior researcher with expertise in qualitative and mixed methods studies was invited to facilitate the session with young partners to elicit their perspectives of how they viewed the data. Drafts of the developing codes, categories, and themes supported by key quotes were shared with young partners to ask their perspectives and thoughts of whether the information made sense or could use further clarification, and whether there was further information they would like to have asked participants. Overall, young partners could be guided to share their perspectives of how they interpret the study data.

#### Implementation phase

During the implementation and knowledge translation activities of the study, all projects provided opportunities for young partners to be involved with co-presentations at national and international conferences. Young partners of the CFP Youth Panel spoke to key persons in politics, sciences, and societal organizations to improve the position of young people with disabilities, using the outcomes of the projects of the CFP program. Young partners in the BEST SIBS Study shared their personal stories and motivations for partnering in research to raise awareness about the important roles that siblings have in all aspects including research. Young partners of the VIPERS Project provided recommendations to central government, local government, strategic managers, and services, while young partners of the RIP:STARS Project presented to stakeholders and responded to government consultations. In addition to co-presentations, young partners from the VIPERS Project, RIP:STARS Project, READYorNot™ BBD Project, PiP Project and the BEST SIBS Study had the opportunity to co-author publications with researchers [36–40]. For projects that have concluded, young partners could inform the next project. For example, the young partners of the VIPERS Project subsequently developed the idea and concept for the research that later became undertaken by young partners in the RIP:STARS Project, which helped to carry on the legacy of the work by the VIPERS.

In each project phase, most teams formed subgroups based on the activities that young people expressed an interest in. The length of time that young partners have been involved in projects is illustrated in Additional file 5. Some projects, for example, the PiP Project and VIPERS Project, had the same young partners involved throughout the project; other projects, for example, the BEST SIBS Study, CFP Youth Panel, READYorNot™ BBD Project, and RIP:STARS Project had new young partners join or other young partners take a step back when needed during the project. All teams had researchers and young partners working collaboratively together to highlight each other’s strengths during certain project activities and support the interests of all members. The RIP:STARS and VIPERS Projects took a further step in explicitly stating that they operated within the social model of disability, in which they collectively addressed any barriers to ensure that all members had an opportunity to be involved with the project activities.

### Support

Supports were provided to the teams in a variety of ways, including the accessibility of the meetings, compensation, and dedicated staff to support the whole team. Details about the supports offered by teams are provided in Additional file 4. For earlier case study projects that were conducted before the COVID-19 pandemic, such as the CFP Youth Panel, PiP Project, and VIPERS Project, there were in-person meetings. The locations were selected based on their accessibility, including being wheelchair accessible or providing quiet spaces for time out. Other individual needs were considered, for example, the CFP Youth Panel ensured that a resting room and allergy considerations were taken into account. While there was aimed to anticipate on individual needs of young partners, such as resting rooms, the project teams emphasize that they would have wanted more time and resources to take these personal desires more into account.

The format of the meetings was influenced by the context of when the projects began. Some projects were conducted prior to the COVID-19 pandemic and were able to conduct a combination of in-person and virtual meetings. Some teams, such as the PiP Project and RIP:STARS, had a combination of meetings that in-person or teleconference (e.g., Skype). While some meetings occurred on a regular basis, the VIPERS and RIP:STARS Projects had meetings that took place for the full day in-person which provided time for socializing, and in-depth training about research and delivery of the project. Through reflection, the day was scheduled to include defined periods of ‘work’ delivered through creative methods of approximately 45 min, followed by a break, and a lunch break where we could share food and socialise together. Young people reflected positively on the in-person meetings, and wanted more of them. Other projects were conducted during the COVID-19 pandemic and only had their meetings online. For example, meetings in the BEST SIBS Study and READYorNot™ BBD Project only took place online through Zoom, which a toll-free number was provided to ensure that young people could attend the meeting at no additional cost for the call to them.

Compensation was provided to young people involved in partnering with researchers on the team. The funds that were used towards compensation came from different sources that were available in the specific country. For example, the CFP Youth Panel and PiP Project received funding from the FNO which was an organization that supported initiatives to increase opportunities for people in the Netherlands. The VIPERS and RIP:STARS Project received funding from The National Lottery in the United Kingdom. In Canada, the Canadian Institutes of Health Research (CIHR) has a Strategy for Patient-Oriented Research (SPOR). The READYorNot™ BBD Project was awarded funding from this institute with partner funding. The BEST SIBS Study was a doctoral research study, and while there was no funding for partner compensation at the beginning, young partners and the doctoral student (LN, first author on this paper), partnered together to submit grants and received two awards, from CIHR and the CHILD-BRIGHT Network (funded by SPOR).

Details about the funding received by each case study project are described in Additional file 1.

There were a variety of forms of compensation. Some teams offered an annual honorarium fee with additional compensation for involvement in activities, and the BEST SIBS Study and READYorNot™ BBD Project offered compensation based on guidelines offered by the CHILD-BRIGHT Network [41]. For teams that had in-person meetings, the travel costs and meals were covered. Some teams incorporated social activities that were covered financially, for example, young partners in the PiP Project attended a museum or cooking workshop. Further details about compensation are provided in Additional file 4. While compensation supported the involvement of young partners in research, we reflected about the importance of asking young people about how they wish to be compensated. Young partners described how they preferred to have some of the funds for compensation used for team activities. The team activities helped to build rapport between young people and the researchers, which was a strength of the teams. Examples of team activities included attending day trips or workshops together, sending e-gift cards sent to order meals and meet virtually, or sending care packages. Young people appreciated these social activities and would have wanted more of these kinds of activities to get to know their team members. Young partners reflected on the importance of remembering to be human with time for fun and laughs through these activities and even during the meetings throughout our partnership.

An important component to continue to build and sustain partnerships with young people is having dedicated staff to facilitate team activities. Some projects had one or two research coordinators for the team, who could be a dedicated individual hired for the project, researchers, or graduate students. The CFP Youth Panel had a member of the panel who was hired to be the chair a part-time job for 8–13 h a week, with support from other members of the team including the program leader and support officer of the CFP program. Some teams reflected on opportunities to have more young people partnered on the projects, but there would need to be considerations about availability of resources such as funding for compensation and personnel support.

### Communication

Supports for regular and proactive communication were a key aspect to ensure that all team members were informed of the project activities. There was clear communication about the steps and purposes, and their involvement, which included invitations, in meetings, and constant contact in between meetings. All teams were proactive in continuing their communication using asynchronous modes outside of meetings, such as by sending regular updates by email and reminders for upcoming meetings. Some young people preferred to receive their updates and communications from the research team on other platforms, such as WhatsApp or Facebook messenger. Private group networking platforms were also used to connect as a team, including a platform called “Notebook” for the PiP Project, and a Facebook group for the BEST SIBS Study and READYorNot™ BBD Project. In all projects, young partners felt that the communication platforms were accessible and tailored to their needs. The variety of communication platforms allowed young partners to choose how they would like to be involved and informed about the projects. Young partners appreciated the flexibility to share their experiences, such as by emails, during team meetings, or during individual check-in meetings.

In addition to communication platforms, documents could be provided as a reference for young people and these documents could be a work-in-progress to be revisited throughout the project. For example, young partners in the BEST SIBS Study reflected that they would have liked to have documents including: Terms of Reference that outlined the project descriptions, possible roles and responsibilities to be discussed with young partners, and forms of compensation; Group Rules to describe expectations during meetings and on the Facebook group; and Activity Log that described the types of activities and hours contributed to the activities. Young partners in the BEST SIBS Study reflected on how information from the Activity Log would be helpful to include on a resume for their professional development. Based on feedback from the research team and young partners, these documents were created later on in the project.

A unique aspect of communication is the personal characteristics of the coordinator for each team. Specifically for young partners in the BEST SIBS Study, the member composition consists of young adults exclusively, including the graduate student researcher who was the facilitator of the group. This member composition of young adults allowed for a unique vulnerability and openness of conversation during meetings about the sibling experience. For the CFP Youth Panel, young partners could connect and communicate directly with the chair, who acted as a liaison to communicate with the other members of the CFP program team. The chair was a young partner with experience who helped to improve communication as members of the CFP Youth Panel and chair understood each other and were in similar phases in life. The author team reflected that the coordinators of all projects had personal traits that contributed to the successful partnership between the researchers and the young people: flexibility, openness, patience, willingness to listen, and a conscientiousness for everyone’s knowledge base (e.g., explaining and clarifying information while avoiding the use of acronyms). The coordinators made the effort to consistently communicate about the value that young people brought to the research projects.

### Proportional

Throughout the process of involving young people in research, the teams aimed to tailor the needs of all members of the team including the young people themselves while balancing the research demands and available resources. All teams had meetings, where young partners had conversations with researchers, project leaders, or facilitators about the partnership; young partners were asked the roles that they would like to have in the projects and in various activities. In the VIPERS and RIP:STARS Projects, young partners were co-leaders and all young people decided together about the level of involvement that each person would like to have at each stage of the research cycle. They had different roles, such as undertaking fieldwork or planning a conference. These conversations took place during in-person meetings. In some projects, there was a chair or coordinator who young people could connect with to communicate about their level of involvement or changing involvement. Some projects also had communication tools that helped to facilitate the conversation with young people. In the PiP Project, the group had regular discussions about roles in next steps and activities. Young partners in the PiP Project later recognized the importance of discussing roles and expectations, which led to the co-development of the Involvement Matrix [26]. The Involvement Matrix could be used as a conversation tool to discuss the roles that young people would like to have in research (e.g., listener, co-thinker, advisor, partner, or decision-maker) for tasks in different stages of preparation, execution and implementation in the research project [26]. Other teams, specifically in the BEST SIBS Study and READYorNot™ BBD Project, were formed after the development of the Involvement Matrix and used this tool along with other tools to have conversations about the roles of young people on the team. Both teams with the BEST SIBS Study and READYorNot™ BBD Project had regular check-in meetings, which they used the Start, Stop, and Continue activity (i.e., what activities the team should start doing, stop doing, or continue doing) and the Involvement Matrix [26], while the team with the BEST SIBS Study additionally used the Patient Engagement Tool from the Ontario Brain Institute to identify examples of how young partners can be involved in research at different stages of the project (e.g., plan the study, recruit and retain participants, do the study, analyze the results, and/or disseminate the results).

### Capacity building

Capacity building relates to the co-learning between young people and researchers, with opportunities for training and learning by experience. While most projects did not offer formal research training, there were considerations from the researchers about how to explain research concepts to young people. In all projects, young partners were explained about the different concepts of research. However, some young people, such as the young partners in the PiP Project, reflected that they would have appreciated opportunities for training about research. Young partners in the READYorNot™ BBD Project reflected about how they would have wanted more conversations with researchers about the terms of “patient-oriented research” and “co-design” to understand how each team member understood these terms and how team members can help each other learn and conduct the project in partnership. Some young people may have had training prior to joining a research project, specifically around the meaning of patient-oriented research. For example, a young partner in the BEST SIBS Study completed the Family Engagement in Research course offered by McMaster University, CanChild Centre for Childhood Disability Research and the Kids Brain Health Network [42]. Some teams offered training if young people expressed they had an interest for training about specific aspects of research.

In the CFP Youth Panel, young partners had the opportunity to be trained in specific activities of the project that they were interested in, such as political activities, conversation strategies, and social media and communication workshops. Young partners of the CFP Youth Panel were also offered a year-long program about advocacy. They were also supported in activities that were specific to the research project. In the VIPERS and RIP:STARS Projects, young partners were encouraged to take on more leadership roles, and they were trained for these roles which included presentation skills, engaging with media, and learning how to budget. Training was also offered to young partners in the VIPERS and RIP:STARS Projects that followed the same pathway and knowledge as an academic level research methods course at the university level but adapted to make it accessible to the individual needs of the group.

For all projects, as young partners reflected on how they gained confidence in their knowledge and skills, they took on the role of being leaders on the project such as being chair of a subteam in the CFP Youth Panel or co-presenting at international conferences for the BEST SIBS Study, PiP Project, READYorNot™ BBD Project. Upon reflection with young partners, researchers recognized the value of learning from and together with young partners about topics that were important to address in the research projects. In the VIPERS and RIP:STARS Projects, the project had an iterative approach to ensure that there was bidirectional learning between researchers and young partners. This ongoing bidirectional learning ensured that the projects were co-led with young partners. Such reflective practice led the RIP:STARS Project team to theorise and publish on a number of tensions between disabled young people becoming research leaders and dominant ideas about disabled children in a disabling society such as overprotection, being empowered through engagement within the project yet restricted in other areas of their personal life, and the emotional impact on disabled young researchers of gathering evidence of a continuing lack of autonomy and rights-based provision for disabled children and young people [43].

## Recommendations

As an author team, comprised of both researchers and young partners, we reflected about strategies of how we partnered together. In this section, we present ‘calls to action’ for teams who wish to form partnerships between researchers and young people, which are based on our experiences, reflections, and lessons learned. While these calls to action are applicable to partnerships with stakeholders, there should be additional considerations when partnering with young people in research [19, 44].

1. *Remember that it is okay to not know what the partnership might look like and there is no single recipe of how to partner with young people* There are different models to involve young people in research, as illustrated by our case study project teams. The roles of young people can also change over time. For example, young partners in the CFP Youth Panel were initially involved as advisors to advise the CFP program and over time, and they also became an advocacy and expert group. Young people may have opportunities to be involved as partners from the start and throughout the projects, as illustrated by young partners involved with the PiP Project, VIPERS Project, and RIP:STARS Project. Young people may also be partners on projects in a council comprised of both young people and parents/caregivers along with researchers, for example, the model of the PFAC with the READYorNot™ BBD Project. Young people may represent their perspectives in their roles as part of the family of an individual with a disability, such as the role of siblings with the SibYAC with the BEST SIBS Study.

The case study project teams all used a variety of strategies to involve young people in research. Some key strategies that were similar across all case study project teams, which included having supports to ensure the accessibility of the meetings that were in-person and virtual, offering compensation in different formats, and having coordinators to facilitate successful partnerships between researchers and young people. There should be flexible and proactive communications, which offer opportunities for young people to be involved in the projects. Young people could be involved in projects by providing feedback during project team meetings or asynchronously by email. The project might evolve over time and there may be small groups that are formed to work on specific initiatives that young people are interested in. These small groups may also provide further opportunities for young people to connect and get to know each other. There should also be communication that is proportional to the level of involvement that young people would like to have, and conversations are important to discuss roles and expectations with young people. These research projects should have opportunities for capacity building with co-learning between young people and researchers.

2. *Take the time to invest and build rapport in partnerships* Partnerships are an investment in knowledge and skills that have valuable benefits for the research projects, young people, and researchers. It is important to take the time to get to know young people, including their motivations, interests, and personal goals for being involved with the project. The characteristics of the facilitator for each project team was important for building rapport with young partners. Furthermore, the facilitators of all projects had personal traits that supported the building of rapport in the partnerships, including being flexible, open, patient, willing to listen and conscientious of the strengths and skills of young partners. While compensation was provided, young partners also appreciated social activities to build rapport within the team such as attending day trips or receiving care packages. Opportunities were provided to young people to be involved at different stages of the research project. Partnerships with young people is an investment, and project teams reflected about the opportunities provided to young partners to receive training for skill development; for example, attending workshops about political strategies, conversation strategies, and social media and advocacy. In all projects, the time invested in building the partnerships was a facilitating factor that allowed young partners to gain confidence in their knowledge and skills that ultimately led them to take on leadership roles.
3. *Provide ongoing opportunities for the team of researchers and young people to reflect on their partnership experiences* Young partners and researchers valued the opportunity to reflect on their partnership experiences both during the project and while writing this paper. In some projects, young people had the opportunity to reflect and evaluate the partnership including what was working well, how things could be better, and to ensure that everyone felt fully involved in all aspects of the project at the level they choose at that time. Each case study project team chose when to have these reflections and evaluations, which were often at the end of each meeting or stage of the research cycle. However, many young partners preferred to have regular check-in meetings throughout the project. Tools could also be used to have conversations for these reflections and evaluations. For example, young partners in the BEST SIBS Study and READYorNot™ BBD Project reviewed the Involvement Matrix [26] to see whether young people would prefer a change in roles or level of involvement. The SibYAC also provided the Public and Patient Engagement Evaluation Tool [45] at the end of each stage of the research cycle, which provided an opportunity for the SibYAC to anonymously provide an evaluation of the partnership experiences. These reflections provide an opportunity to incorporate feedback to enhance the partnership experiences. Young people may also reflect on the impact of their involvement and contributions in research, which may empower them to take the lead on research initiatives. As young partners and researchers on our author team reflected on the partnership experiences after the project was conducted, we would have liked to have included more opportunities to internally evaluate our partnership experiences including reflections about what was or was not working well throughout the research projects. As a collective team, we recognize how we are learning on the go, in which young people and researchers are learning from each other and create a balance of reflective exercises while meeting the needs of the project.
4. *Consider how to balance the power dynamics in the partnership* There should be considerations about how to reduce the power imbalances between young partners and researchers, which includes the decision-making process in the projects. From the beginning and throughout the project, conversations should be held between young people and researchers, possibly with the help of tools. In the RIP:STARS and VIPERS Project, there was an expectation from the beginning that power was to be a shared concept, which involved the researchers being prepared to give up power and take a step into not knowing how the project and partnership might look like. Tools have also been co-developed through the Family Engagement in Research course [42] for self-reflections between young people and researchers to address power imbalances [46]. While there may be constraints about certain decisions that are made, for example, from the funding agencies or institutions, there may be other initiatives related to the projects that may provide opportunities for young people to feel empowered and become decision-makers [43]. For example, young partners from the CFP panel took the initiative to not only provide advice for projects but to also suggest new projects that were not covered by the grant applicants including setting up a project about a platform to share experiential stories. Similarly, the SibYAC also suggested expanding the website to describe both the project’s work and also blogs about their stories as siblings of youth with disabilities, and some young partners of the PiP Project decided to build a website for young persons with cerebral palsy as part of a knowledge translation initiative. As the partnership developed over time, the power shifted in which young people began to take on leadership roles for initiatives related to the research project. Partnership includes making sure that young partners feel valued, respected, and seeing that their involvement makes a difference to the project. It is important for projects to be open to allow for the change in partnership dynamics.
5. *Consider how to incorporate diversity in the background of young partners involved in research* It is important to consider the diversity of young partners who have the opportunity be involved in research. Some projects teams, such as the BEST SIBS Study, READYorNot™ BBD Project, and the RIP:STARS project, are continuing their partnership and will have an opportunity to recruit additional partners from diverse backgrounds. For example, young partners with the BEST SIBS Study have considered how to recruit young partners from different genders and ethnicities moving forward. While the recruitment of partners from hard-to-reach populations can be challenging, different strategies can be used such as working with community organizations to recruit these partners [47]. Furthermore, the initial partnership could begin with one or two young partners and continue to expand over time, similar to how the partnership with young partners with the BEST SIBS Study began which started with two young partners and has expanded to have six young partners at the time of this paper. For young people who might not have had as much experience with partnerships in research, there could be opportunities for mentorship. For example, reflections from the READYorNot™ BBD Project identified there could be a ‘buddy system’, in which experienced partners could be paired with new partners. The experienced partners could answer questions, share resources, or provide feedback and comments about ways that new partners could be involved in the project.

## Conclusion

In this paper, as a team comprised of young partners and researchers, we reflected about our experiences in partnering together in research. Across all six case study project teams, the perspectives of young partners were valued to inform different stages of research. Even throughout the process of writing this paper, we prioritized and asked young partners to share what they had learned. By engaging in this reflection process using the framework outlined by Liabo et al. [25], we identified how our partnerships had an impact on not only the research, but also on young partners and researchers.

In each of our case study projects, we identified similarities and differences in partnerships with young partners in Canada, the Netherlands, and the United Kingdom. While all case study project teams had similar values, there were both similar and different approaches in the practicalities of how we implemented our values. During our reflection process, we also recognized how partnerships can change over time both within a single project team and also across project teams.

The case study project teams were conducted at different times. Some project teams had already completed their projects, and this reflective process was useful to identify their positive experiences and consider what they might have done differently. For project teams that are continuing their partnerships at the time of this writing were able to use this reflection process in this paper and the learnings from other project teams to improve certain areas in their own projects moving forward. As young partners continue to increasingly be involved in research, we hope that the sharing of our lessons learned can be beneficial to current and future research teams. We hope this article is viewed as invitation to expand and exchange knowledge on how to involve young experts by experience in research.

## Supplementary Information

**Additional file 1**. Description of the project and characteristics of young partners involved in the project.

**Additional file 2**. Framework about the values for public involvement in health research.

**Additional file 3**. Involvement in activities.

**Additional file 4**. Framework about the practicalities for public involvement in health research.

**Additional file 5**. Length of time of involvement in projects.

## Data Availability

All data generated or analysed during this study are included in this published article.

## Abbreviations

BEST SIBS Study: BrothErs and Sisters involvement in health care TranSition for youth with Brain-based disabilities Study
CFP: Care and future prospects
CIHR: Canadian institutes of health research
PiP Project: Participation in perspective project
READYorNot™ BBD Project: READiness in Youth fOR transition out of pediatric care brain-based disabilities brain-based disabilities project
RIP STARS Project: Research into practice: skilled team with ambition, rights and strength project
SPOR: Strategy for patient-oriented research
VIPERS Project: Voice, inclusion, participation, empowerment, research project

## Footnotes

1 Six case study projects: 1) Care and Future Perspective (CFP) with the Youth Panel in the Netherlands with EvdM and BvO; 2) Participation in Perspective (PiP) project in the Netherlands with LS and MK; 3) Voice, Inclusion, Participation, Empowerment, Research (VIPERS) project in England with AF; 4) Research into Practice: Skilled Team with Ambition, Rights and Strength (RIP:STARS) Project in England with AF; 5) BrothErs and Sisters involvement in health care TranSition for youth with Brain-based disabilities (BEST SIBS) Study with the Sibling Youth Advisory Council (SibYAC) in Canada with HD and LN; and. 6) READiness in Youth fOR transition Out of pediatric Care Brain-Based Disabilities (READYorNot™ BBD) Project with the Patient and Family Advisory Council (PFAC) in Canada with CDM, LN, and JWG.
