# Supplementary material for: Exploring the “how” in research partnerships with young partners by experience: lessons learned in six projects from Canada, the Netherlands, and the United Kingdom
Source: Res Involv Engagem. 2022 Nov 17;8:62. doi: 10.1186/s40900-022-00400-7 (PMC9672637; doi:10.1186/s40900-022-00400-7)
Supplement: Supplementary file 1 — Additional file 1. Description of the project and characteristics of young partners involved in the project. [file 40900_2022_400_MOESM1_ESM.docx]

**Additional File 1. Description of the project and characteristics of young partners involved in the project.**

|  | **CFP Youth Panel** | **PiP Project** | **VIPERS Project** | **RIP:STARS Project** | **BEST SIBS Study** | **READYorNot™ Brain-Based Disabilities Project** |
| --- | --- | --- | --- | --- | --- | --- |
| **Description** | | | | | | |
| Project Aim | The CFP Youth Panel was founded to advise the CFP program on which projects to subsidise to help young people with a chronic condition. They also advised the chosen projects throughout their duration. The CFP Youth Panel continued to expand its work and influence to improve the social position of young people with chronic disorders in five areas: care, school, work, sport and empowerment. | The PiP Project was a research project aiming to understand the experiences and stories of young experts with the lived experience of CP about participation and autonomy in the context of school, sport and care. | The VIPERS Project was a disabled young people co-led research study which examined the participation of disabled children and young people in decision-making at a strategic level within services across England. | The RIPSTARS Project is a disabled young people co-led research collective which undertakes research studies to inform policy and practice concerning the rights of disabled children and young people both nationally and internationally. | The SibYAC have been involved in the design phase of a qualitative study called the BrothErs and Sisters involvement in health care TranSition for youth with Brain-based disabilities (BEST SIBS) Study, which aims to understand the roles and responsibilities of siblings and have a brother or sister with a diagnosis of a brain-based disability. | The Patient and Family Advisory Council (PFAC) was developed to partner in the  READYorNot^TM^ Brain-Based Disabilities Project, a patient-oriented research project. The project aims to develop and evaluate the effectiveness of the MYREADY Transition^TM^ BBD App to empower youth during their transition from pediatric to adult health care. |
| Number of youths involved during the span of the project | 11-22 youth | 12 youth, called ambassadors | 16 youth, called Vipers. | 6 young people called the RIPSTARS. | 6 young adult siblings, as part of the Sibling Youth Advisory Council (SibYAC) | 8 young people as part of a larger Patient and Family Advisory Council (PFAC) with parents |
| Background of youth | - Ages 15-30  - With all types of chronic conditions such as rheumatism, diabetes, cerebral palsy and traumatic brain injury | - Ages 12‐17 years  - With cerebral palsy | - Ages 12-21 years  - Pan-impairment, in which the group works within a social model of disability so is open to any young person who considered themselves to be disabled | - Currently, ages 15-23 years. - The group is organic, and having secured more funding will allow for the the recruitment of more members aged 12-25 years - Pan-impairment, in which the group works with a social model of disability so is open to any young person who considered themselves to be disabled | - Ages 21-27 years  - Have a brother or sister with a disability, which includes autism spectrum disorder, cerebral palsy, the genetic condition of CDKL5 and multiple sclerosis | - Ages 16 - 38 - With lived experience of a chronic health condition |
| Recruitment of young people to be involved in the project | Youth were invited to join the panel through social media campaigns, projects of the CFP program, one-to-one-contact and school counsellors. | The youth ambassadors were recruited through a call by the patient organisation BOSK and by calls on various social media platforms. | Via voluntary sector organizations and advocacy groups across England. | Originally via a local disability advocacy service. | The siblings were already engaged in the network of CanChild and were asked to join this project by the project student investigator. | Youth and young adults were connected through a variety of approaches, including those who were involved in previous CanChild studies and recruitment advertisements through the CHILD-BRIGHT Network. |
| Organization and/or funding | Organisation and funding: FNO, a fund that supports initiatives to improve opportunities for vulnerable people in the Netherlands. | Organisation: Center of Excellence for Rehabilitation Medicine (UMC Utrecht and De Hoogstraat Rehabilitation) and BOSK.  Funded by FNO, a fund that supports initiatives to improve opportunities for vulnerable people in the Netherlands. | Organisation: Council for Disabled Children, National Children’s Bureau, The Children's Society and the Alliance for Inclusive Education; all non-governmental organizations.  Funded by The National Lottery. | Organisation: Universities of Portsmouth/  Nottingham Trent. We partner with multiple voluntary and statutory organisations depending on current agendas.  Funding by The National Lottery, British Academy, Blagrave Trust. | Organization: CanChild.  Funded by Patient-Oriented Research Award – Transition to Leadership Stream (Phase 1) through the Canadian Institutes of Health Research and the Graduate Fellowship in Patient-Oriented Research through the CHILD-BRIGHT Network. | Organization: CHILD-BRIGHT Network.  Funded by the Canadian Institutes of Health Research Strategy for Patient-Oriented Research with partner funding. |
