# Supplementary material for: Exploring the “how” in research partnerships with young partners by experience: lessons learned in six projects from Canada, the Netherlands, and the United Kingdom
Source: Res Involv Engagem. 2022 Nov 17;8:62. doi: 10.1186/s40900-022-00400-7 (PMC9672637; doi:10.1186/s40900-022-00400-7)
Supplement: Supplementary file 2 — Additional file 2. Framework about the values for public involvement in health research. [file 40900_2022_400_MOESM2_ESM.docx]

**Additional File 2. Framework about the *values* for public involvement in health research** [1]**.**

| **Values** | | | | | | |
| --- | --- | --- | --- | --- | --- | --- |
|  | **CFP Youth Panel** | **PiP Project** | **VIPERS Project** | **RIP:STARS Project** | **BEST SIBS Study** | **READYorNot™ Brain-Based Disabilities Project** |
| **Inclusivity:** Involvement of a diverse range of people and equal opportunities for people to be involved regardless of social background and abilities. | Everyone who was interested had the opportunity to be involved in activities with the panel. No exceptions were made for social background, status or disabilities. People were welcome to join at any time throughout the program. | Everyone who responded to the call to be a young partner with the PiP Project and was willing to be involved after hearing about the explanation of expectations, was welcome. | Anyone who was interested in the call to become a disabled young researcher. | Anyone who was interested in the call to become a disabled young researcher. | All members were welcome to be part of the SibYAC after they heard information about the projects, and had a discussion about roles and expectations. There were equitable opportunities for individuals with different circumstances (e.g., time, responsibilities at home, work and school commitments) to still be involved. | All members were welcome to be part of the PFAC after learning about the project and having a discussion about expectations and roles. |
| **Partnership:** Showing respect for each other’s contributions and roles, and working together as a team. | The efforts of the members of the CFP Youth Panel were appreciated by the CFP program through various ways; for example, (financial) compensation, verbal appreciation, recognition of their work/efforts, opportunities to engage in networking and training, etc.  At the start of the CFP program, the Youth Panel was just one of the two advisory bodies of the CFP program for grant applications while the other advisory body consisted of experts in the field/professionals. However, over time the youth panel had a more assertive role with the chair of the Youth Panel becoming a part of the CFP Program at FNO as a part-time job and the panel became more than an advisory body. They took the lead in certain projects and activities such as political advocacy, which brought up new topics, and their voice was heard throughout the whole program. This iterative change from advisory to leading body was seen as a positive development by all partners. | Appreciation for contributions was verbalized in many ways. The input of young partners was important in the direction and choices during the research.  Both researchers and youth partners felt the input was complementary.  Mostly there was clear communication about what was done with input and feedback.  Young partners felt appreciated. The decision to have a professional team making videos of the youth partners for the website they created was, according to the youth partners, a nice example of how their work was appreciated. These videos also supported youth partners in thinking about their role and commitment, and what to say in their personal video.  An interesting experience for the youth partners was a workshop during a conference of the European Academy of Childhood Disability, where some youth partners shared their experiences; many participants reached out to them to thank and emphasize the importance of their message. | All decision-making was jointly undertaken and we all worked as a team recognising each other's strengths, interests and talents. Individual choice was given as to the level of involvement at each stage of the research cycle; for example, some chose to undertake fieldwork, others preferred ‘behind the scenes’ involvement. Levels of involvement was an ongoing choice as confidence increased, the young partner’s leadership increased. | All decision-making was jointly undertaken and we all worked as a team recognising each other's strengths, interests and talents. Individual choice was given as to the level of involvement at each stage of the research cycle; for example, some chose to undertake fieldwork, others preferred ‘behind the scenes’ involvement. The level of involvement in activities was an ongoing choice. | Appreciation for contributions and the value of partnership was verbalized consistently in meetings and in follow-up communications.  Meeting agendas allowed for time for everyone to express their views and experiences.  Partnership was recognized in different ways; for example, through discussions with special guests in meetings, co-presentations, and transparent updates.  Care packages and virtual gatherings for the holidays were held to celebrate our achievement of milestones for the year.  Agenda for monthly meetings included time for life updates. This check-in functioned as peer support which was a sign of respect for the holistic nature of being a sibling partner. | Appreciation for members' contributions was clearly stated by research staff both verbally and in writing. The research team was open to feedback and criticisms and demonstrated a willingness to change to improve partnerships. Feedback was asked from PFAC members through individual activities with the ‘Start, Stop, Continue’ exercise to discuss: What should we start doing, What should we stop doing, and What should we continue doing?.  There were many opportunities for members to become more involved, and when a young partner signed on to an additional project or subgroup, roles and responsibilities were decided collaboratively. |
| **Purposeful:** Clarity on why young partners are involved in research, which is communicated to everyone involved, commitment to involvement. | In the CFP program, collaboration with the Youth Panel was compulsory for projects, researchers and partners. While some project leaders and partners were not used to this form of collaboration, over time, everybody recognized the need to work together with young people as necessary to achieve their goals and to obtain effective and long-lasting results. “Nothing about us without us” became a standard. However, the way the partnership was organized differed per activity. | The importance of involvement in the ‘why’ was clear, and communicated both in the invitation as well as in the meetings and through contacts in between meetings. Also in external communication the ‘why’ and the impact were communicated. | The values underpinning the project were one of co-leadership and equality across the team. This ethos was shared and explained at the outset of the project. However, an understanding of what this ethos meant in reality grew as the project developed and the ethos was operationalised with real, tangible experiences. | As young partners co-lead the project, they worked with potential partners to ensure that the ethos of co-leading by disabled young people was understood and embraced by whoever they worked with.    Young partners in the RIP:STARS Project now support and/or train other disabled young people to become young researchers and share their own experiences as a way to impart information to others. | There was clear communication of purpose and roles when a potential initiative/collaboration was proposed to sibling partners.  Since January 2021, there was a plan of initiatives for the year with a description and sign-up sheet. Young partners could choose the level of involvement that they would like to have in each initiative. | There was a clear message when sending an invitation to ask PFAC members to be a part of an initiative, including a description, what their involvement would look like, and time commitment. Invitations were asked during the PFAC meetings and by email. |
| **Transparency:** Having open and honest communication between young partners and researchers, providing clarity on why things are done in certain ways. | There were more direct communication lines between the Youth Panel and CFP program, which was especially due to the role of the chair. This form of communication provided many opportunities for young partners to provide input. Moreover, what was found helpful were clear communication moments, such as during meetings or over email. | Open and honest communication was especially felt during meetings.  During the project we realized that in between the meetings, young partners sometimes felt there was a lack of clarity about what was done and why. | Open and honest transparency was vital to develop a team ethos and co-led decision-making process. We adopted multiple, accessible methods and personal and collective reflections throughout to ensure communication, ways and levels of involvement were being delivered and implemented in a way that met any access needs and supported the group to meet their own and VIPER collective goals. For example, materials and our approach to communication and involvement took into consideration that the group's access needs included those with visual and communication impairments, learning disabilities and neurodiversity. Delivery was always in multiple, synchronised ways. | Open and honest transparency is vital to develop a team ethos and co-led decision-making process.    We adopt multiple, accessible methods and personal and collective reflections throughout to ensure communication, ways and levels of involvement are being delivered and implemented in a way that meets any access needs and supports the group to meet their own and collective goals. | Transparency regarding:  Communications with external stakeholders (e.g., committee members, NYAP, guest speakers, granting agencies), ensuring sibling partners are comfortable with arrangements being made and asking if young partners were comfortable being named on initiatives. | There was a shift towards having clear communication by sharing with the PFAC about how their feedback was incorporated, as a method of ‘closing the loop’. |
| **Value different kinds of knowledge:** Recognition that young partners have complementary expertise to researchers’ technical knowledge. | When joining the panel, young partners were asked what topics they found interesting or what kind of expertise/experience they wanted to add to the panel. Based on this they were assigned to one or more ‘theme groups’ (like education, sports, health care, etc.). This way they could provide input based on their expertise. This was recognized and appreciated by the CFP program team, researchers, stakeholders and project leaders. They were often surprised by the knowledge and opinions the young people shared. Mostly, these were opinions or directions the researchers did not realize to be important and sometimes this caused a different approach and direction of the research/project itself. They realized the opinions and input of the target group were essential to make the final outcome/product a success. | In all meetings and all activities, young partners were involved and the value of their perspective was emphasized. Young partners were encouraged to share their perspectives and ideas.  New research topics emerged from the input of young partners, and both researchers and ambassadors felt that combining expertise has led to valuable ideas, activities and products. | In both projects, there was an assumption that all knowledge and experience is valid. The project aimed to make the ‘technical’ language and undertaking of research accessible to young disabled people; thus dismantling the notion that only academics can undertake rigorous and valid research.  Utilising accessible language, and with guidance and support, the group successfully undertook academic level research. Their training for example, followed the same pathway as an academic level research methods course at university level. | | Young partners were engaged at all stages of the research process.  Meetings include updates of study progress, and iterative solicitation of feedback to improve recruitment methods. Feedback could also be provided by emails or individual check-in meetings.  At conference and poster presentations, young partners were encouraged to share their perspectives and experiences. There were 1-2 planning meetings prior to the presentations to discuss questions and preparation of content.  Partners were all young adults - including the facilitator - which allows for a unique vulnerability and openness of conversation during meetings about the sibling experience. This led to creative and tailored KT initiatives: social media campaigns, podcasts and blogs. | The Involvement Matrix was used as a conversation tool to guide the discussion during check-in meetings with each partner. The use of the Involvement Matrix highlights that any level of commitment is important, as lived experience is extremely valued in the project. |
| **Evaluation:** Identifying good practice, through communication, research and learning from each other. | For young partners, it was very important to know what happened with their input and feedback. Within the panel and the CFP program, it was aimed that young partners always heard what the results of the collaboration were. Young partners were able to provide FNO and/or the chair with feedback and new ideas to improve communication and working together.  The program and the panel were evaluated by an independent research institute to identify success indicators, challenges , results and recommendations. This report also included a section with tips for project leaders/researchers to work with youth and tips for youth to participate in research and projects. This was published at the end of the CFP program (4 years since the start of the program/panel). | There were no formal moments of evaluation, taking a step back.  During the project, there were some natural situations that supported reflection and learning.  Based on these reflections, it was decided to develop a tool to support collaboration and to support the conversation about roles and expectations, the Involvement Matrix [2,3]. | At the end of each meeting and stage of the research cycle, young partners undertook self-evaluation to reflect on what had worked, how we could be doing things better, to ensure we were fully inclusive and that everyone felt fully involved in all aspects of the study at the level they chose at that time.  An independent evaluation of the study, and its co-led methodology was also undertaken to facilitate open, honest feedback and learning. The evaluation also examined the impact of the project on the young researchers and the external outcomes of this disabled young person co-directed research study. The impact on the young people included increased confidence, self-esteem, sense of achievement, new skills which supported their education and further personal development. Some reported using this experience and skill development to secure employment, higher and further education courses. | At the end of each meeting and stage of the research cycle, young partners undertake self-evaluation to learn how we could be doing things better, to ensure we are fully inclusive and that everyone feels fully involved in all aspects of the study at the level they choose at that time.  At the end of each project, we assessed what worked and how we can improve. | Open lines of communication for feedback (email, meetings). Monthly check-in/update meetings  Annual evaluation of the partnership experience: one-on-one meeting, Public and Patient Engagement Tool [4], Involvement Matrix [2] and re-evaluating roles. | Individual check-in meetings were held biannually with each PFAC member with two activities:   1. Start, Stop, Continue activity, where feedback is provided about how we are doing as a PFAC as well as changes that would like to be implemented. 2. Involvement Matrix [2]   These activities allowed us to reflect on our partnership and have feedback from the PFAC. |

**References**

1. Liabo K, Boddy K, Bortoli S, Irvine J, Boult H, Fredlund M, et al. Public involvement in health research: what does ‘good’ look like in practice? Res Involv Engagem. 2020;6:11.

2. Smits D-W, van Meeteren K, Klem M, Alsem M, Ketelaar M. Designing a tool to support patient and public involvement in research projects: the Involvement Matrix. Res Involv Engagem. 6:30.

3. Center of Excellence for Rehabilitation Medicine Utrecht. The Involvement Matrix [Internet]. [cited 2022 May 26]. Available from: https://www.kcrutrecht.nl/involvement-matrix/

4. Abelson J, Tripp L, Kandasamy S, Burrows K. Supporting the evaluation of public and patient engagement in health system organizations: Results from an implementation research study. Heal Expect. 2019;22:1132–43.
