# Supplementary material for: Exploring the “how” in research partnerships with young partners by experience: lessons learned in six projects from Canada, the Netherlands, and the United Kingdom
Source: Res Involv Engagem. 2022 Nov 17;8:62. doi: 10.1186/s40900-022-00400-7 (PMC9672637; doi:10.1186/s40900-022-00400-7)
Supplement: Supplementary file 3 — Additional file 3. Involvement in activities. [file 40900_2022_400_MOESM3_ESM.docx]

**Additional File 3. Involvement in activities.**

|  | **Youth Panel CFP** | **PiP Project** | **VIPERS** | **RIPSTARS** | **BEST SIBS Study** | **READYorNot™ Brain-Based Disabilities Project** |
| --- | --- | --- | --- | --- | --- | --- |
| **Involvement in** PENCRU research cycle [1] | | | | | | |
| Idea/concept | ✓ |  | ✓ | ✓ |  |  |
| Research question | ✓ | ✓ | ✓ | ✓ | ✓ |  |
| Prioritising | ✓ | ✓ | ✓ | ✓ | ✓ |  |
| Planning/designing |  | ✓ | ✓ | ✓ | ✓ | ✓ |
| Seeking funding | ✓ |  |  | ✓ | ✓ |  |
| Collecting data | ✓ | ✓ | ✓ | ✓ | ✓ | ✓ |
| Analysing the data |  | ✓ | ✓ | ✓ |  | Not yet started/planned |
| Interpreting the results/drawing conclusions |  | ✓ | ✓ | ✓ |  | Not yet started/planned |
| Telling people the results | ✓ | ✓ | ✓ | ✓ |  | ✓ |
| Changing practice | ✓ |  | ✓ | ✓ |  | Not yet started/planned |
| Re-evaluation |  | ✓ | ✓ | ✓ |  | Not yet started/planned |
| **Examples of Roles and Activities** | | | | | | |
| Preparation | - Reviewed plain language summaries of grant applications - Thinking along with recruitment strategies for both the panel and the chosen projects | - Partnered in developing interview method and interview topics - Partnered in drafting information letter for recruitment | - Partnered in deciding on the overall research question and design to garner funding and co-led all other aspects of the research study - Reviewed and rated potential case-study organizations/   services to further research | - Designed research studies, developed research proposals and sought funding - Developed and delivered research training for disabled children and young people | - Co-developed the research question and supported the identification of study methods - Reviewed grant applications | - Co-developed the App - Prepared e-learning modules to train research assistants |
| Execution | - Gave advice to the CFP program on youth topics - Requested support and later additional funding for project, implementation and policy change | - Partnered in analyses of interviews - Partnered in interpretation of findings | - Undertook all fieldwork for case studies including interviewing professionals and young people - Implemented ongoing evaluation and contributed to an external evaluation of the project | - Undertook all aspects of the research cycle, including the collection and analysis of data - Co-wrote the report and developed recommendations for policy and practice | - Piloted the interview guide - Recruited participants | - Reviewed and provided feedback on the interview guide and data collection forms |
| Implementation | - Spoke to key persons in politics, sciences and societal organizations on improving the position of the target group - Co-presented at conferences | - Four ambassadors presented their experiences at the international congress of the European Academy of Childhood Disability - The ambassadors built a website to share knowledge and findings with peers | - Co-wrote a research report, and guidance tools. Published findings in numerous practitioner publications - Designed, planned and delivered a national launch conference | - Presented and lobbied stakeholders - Invited by the UK government to present their findings at a cross-parliamentary enquiry into provision for special educational needs and disability in England. Their evidence was highly quoted in the parliamentary report. | - Co-presented at conferences | - Co-presented at conferences - Co-created knowledge translation products |
| **Two examples of**  **outputs** | Publications:   1. van Schelven, F., Boeije, H., Inhulsen, M. B., Sattoe, J., & Rademakers, J. (2021). “We know what we are talking about”: Experiences of young people with a chronic condition involved in a participatory youth panel and their perceived impact. Child Care in Practice, 27(2), 191-207. doi:10.1080/13575279.2019.1680529 [2]   Website:  <https://www.fnozorgvoorkansen.nl/afgeronde-programmas-fno/zorg-en-perspectief/resultaten-zep> [3] | Publication:   1. Wintels, S. C., Smits, D. W., van Wesel, F., Verheijden, J., Ketelaar, M., PERRIN PiP Study Group, ... & Gorter, J. W. (2018). How do adolescents with cerebral palsy participate? Learning from their personal experiences. Health Expectations, 21(6), 1024-1034. doi:10.1111/hex.12796 [4]   Website: [www.wijencp.nl](http://www.wijencp.nl) [5] | Book Chapter   1. Franklin A and Todd Z. “They still need to listen more”: Working in partnership with disabled young researchers to inform and shape country submissions to the UN Committee on the Rights of Persons with Disabilities and UN Committee on the Rights of the Child in Beckett A and Callus, A. International Handbook of Children’s Rights and Disability. Routledge, London; 2021 (in press) [6]. 2. Office of the Children’s Commissioner ‘They still need to listen more’: A report about disabled children and young people’s rights in England [Internet]. 2014 [Accessed on 11 May 2022] <https://www.childrenscommissioner.gov.uk/report/they-still-need-to-listen-more/> [7] | Publication:   1. Brady G, Franklin, A. "Challenging dominant notions of participation and protection through a co-led disabled young researcher study", Journal of Children's Services. 2019;14(3):174-185. doi:10.1108/JCS-03-2019-0016 [8] 2. RIP:STARS., Franklin A, Brady, G, and Durell S. Defining Quality and Rights Based Education, Health and Care Plans (EHCPs) for Disabled Children and Young People [Internet]. Coventry: Coventry University; 2018 [Accessed on 1 May 2022] Available from<https://ripstars.net/> [9] | Publications:   1. Nguyen L, Davis H, Bellefeuille S, Havens J, Jack SM, Di Rezze B, Ketelaar M & Gorter JW. Canadian resources for siblings of youth with chronic health conditions to inform and support with healthcare management: A qualitative document analysis. Frontiers in Rehabilitation Sciences: Families and Functioning in Childhood and Adolescence issue. 2021:52. doi:10.3389/fresc.2021.724589 [10]   Website:   1. <https://www.canchild.ca/en/research-in-practice/current-studies/brothers-and-sisters-involvement-in-health-care-transition-for-youth-with-brain-based-disabilities-best-sibs-study> [11] | Publication:   1. Gorter JW, Amaria K, Kovacs A, Rozenblum R, Thabane L, Galuppi B, et al. CHILD-BRIGHT READYorNot Brain-Based Disabilities Trial: protocol of a randomised controlled trial (RCT) investigating the effectiveness of a patient-facing e-health intervention designed to enhance healthcare transition readiness in youth. BMJ Open. 2021;11:48756. doi:10.1136/bmjopen-2021-048756 [12]   Knowledge translation products, including a stakeholder engagement video and research video series, are posted on the study website: <https://www.child-bright.ca/readyornot/> [13] |

**References**

1. PenCRU & Family Faculty, University of Exeter Medical School. What do we mean by research? [Internet]. [cited 2021 Jun 8]. Available from: https://www.pencru.org/research/whatdowemeanbyresearch/

2. van Schelven F, Boeije H, Inhulsen M-B, Sattoe J, Rademakers J. “We know what we are talking about”: Experiences of young people with a chronic condition involved in a participatory youth panel and their perceived impact. Child Care Pract. Routledge; 2021;27:191–207.

3. FNO. Resultaten Zorg én Perspectief [Internet]. 2021 [cited 2022 May 26]. Available from: https://www.fnozorgvoorkansen.nl/afgeronde-programmas-fno/zorg-en-perspectief/resultaten-zep/

4. Wintels SC, Smits DW, van Wesel F, Verheijden J, Ketelaar M, van der Leest A, et al. How do adolescents with cerebral palsy participate? Learning from their personal experiences. Heal Expect. 2018;21:1024–34.

5. CP Nederland, PiP ambassadors. WijenCP [Internet]. [cited 2022 May 26]. Available from: https://www.wijencp.nl/

6. Franklin A, Todd A. “They still need to listen more”: Working in partnership with disabled young researchers to inform and shape country submissions to the UN Committee on the Rights of Persons with Disabilities and UN Committee on the Rights of the Child. In: Beckett A, Callus A, editors. Int Handb Child Rights Disabil. London: Routledge; 2022.

7. Office of the Children’s Commissioner. “They still need to listen more”: A report about disabled children and young people’s rights in England. 2014.

8. Brady G, Franklin A. Challenging dominant notions of participation and protection through a co-led disabled young researcher study. J Child Serv. 2019;14:174–85.

9. RIP:STARS, Franklin A, Brady G, Durell S. Defining Quality and Rights Based Education, Health and Care Plans (EHCPs) for Disabled Children and Young People [Internet]. 2018 [cited 2022 May 1]. Available from: https://ripstars.net/

10. Nguyen L, Davis H, Bellefeuille S, Havens J, Jack SM, Di Rezze B, et al. Canadian resources for siblings of youth with chronic health conditions to inform and support with healthcare management: A qualitative document analysis. Front Rehabil Sci. 2021;2:52.

11. CanChild Centre for Childhood Disability Research. BrothErs and Sisters involvement in health care TranSition for youth wIth Brain-based disabilitieS (BEST SIBS) Study [Internet]. 2021 [cited 2022 Feb 4]. Available from: https://canchild.ca/en/research-in-practice/current-studies/brothers-and-sisters-involvement-in-health-care-transition-for-youth-with-brain-based-disabilities-best-sibs-study

12. Gorter JW, Amaria K, Kovacs A, Rozenblum R, Thabane L, Galuppi B, et al. CHILD-BRIGHT READYorNot Brain-Based Disabilities Trial: Protocol of a randomised controlled trial (RCT) investigating the effectiveness of a patient-facing e-health intervention designed to enhance healthcare transition readiness in youth. BMJ Open. 2021;11:48756.

13. CHILD-BRIGHT READYorNot^TM^ Project Study Team. READYorNot(TM) Brain-Based Disabilities Project [Internet]. 2021 [cited 2022 Feb 4]. Available from: https://www.child-bright.ca/readyornot
