# Supplementary material for: Exploring the “how” in research partnerships with young partners by experience: lessons learned in six projects from Canada, the Netherlands, and the United Kingdom
Source: Res Involv Engagem. 2022 Nov 17;8:62. doi: 10.1186/s40900-022-00400-7 (PMC9672637; doi:10.1186/s40900-022-00400-7)
Supplement: Supplementary file 4 — Additional file 4. Framework about the practicalities for public involvement in health research. [file 40900_2022_400_MOESM4_ESM.docx]

**Additional File 4. Framework about the *practicalities* for public involvement in health research** [1]**.**

|  | **CFP Youth Panel** | **PiP Project** | **VIPERS Project** | **RIP:STARS Project** | **BEST SIBS Study** | **READYorNot™ Brain-Based Disabilities Project** |
| --- | --- | --- | --- | --- | --- | --- |
| **SUPPORTS** | | | | | | |
| **Location of Meeting** | Location was suited and accessible for wheelchair-users. Individual needs such as allergy-proof food were available. | There were online and physical meetings. Location of physical meetings was suited and accessible for wheelchair-users.  Online meetings were held through Skype or teleconference | The group was UK based but travelled to London for face-to-face meetings during weekends/school holidays. These were full day meetings which also gave time for socialising together. Individual access needs were met. | The group met face-to-face each month. During COVID, there were Zoom meetings; however, young partners preferred to meet physically and found online meetings did not meet all of their access needs or enable them to socialise with each other, which was considered an important element to their work. | Monthly meetings were held on Zoom with a toll-free number. | Both full team meetings and subgroup meetings were held through Zoom with a toll-free number. |
| **Compensation** | Young partners were offered a volunteer fee per attended meeting and/or activity (at minimum €300 per year). The panel members were paid €50 per meeting and €25 per external activity (in 2019). All travel costs (also taxi costs when public transport was hard) were covered. | Compensation was provided for specific activities depending on the role. Costs for social activities (e.g., attending a museum, cooking workshop) and meals were covered by the project budget. | Each young partner was given a £10 voucher per meeting and vouchers for days spent undertaking fieldwork. All travel, hotel and food costs were paid for. Costs for some social activities were also paid for. | Wherever funding allows, young partners were compensated via gift vouchers. Costs for social activities are also paid for. All travel and food costs are covered. | Compensation was provided that was guided by the CHILD-BRIGHT Network, and modified based on availability of funding.  Young partners were provided with an annual honoraria of $250 CAD for attending at least 3 meetings within a 6-month period. Additional honoraria was provided for involvement as a partner in initiatives that range from $25-$75. The additional honoraria could also be used towards a group activity instead (e.g., virtual social gathering). | Compensation was provided based on the guidelines outlined by the CHILD-BRIGHT Network [2]. Partners received an annual honorarium of $500 CAD and an honoraria was provided for additional activities, such as the preparation and delivery of a presentation for $100 CAD or participation in a panel for $50 CAD. |
| **Dedicated staff** | One of the young partners of the panel was chair, which was a part-time job (paid by the CFP program of FNO) for 8-13 hours a week. The program leader, secretary and support officer of the CFP program were also closely involved. All contact was directed through and by the chair. | Coordinators of the patient organisation BOSK facilitated the meetings.  Researchers, ambassadors, and coordinators of BOSK were part of a group in social media (a platform decided by young partners). Individual needs were checked before and during meetings by the coordinators of BOSK and researchers. | Alongside the research team, the project had a part-time participation worker to support the young partners and ensure all of their access needs were met. | Two academics supported the day-to-day running of the collective. When funding allowed, additional staff were employed to support intense research activity. | A PhD Candidate (LN, first author of this paper), who was also a graduate research assistant on the READYorNot™ BBD Project and has experience in facilitating patient and family advisory council meetings, was the facilitator of this group. | There was a research coordinator dedicated to the facilitation of PFAC activities and meetings. |
| **Capacity building:** Relates to co-learning between young partners and researchers, and training for both groups.  Training opportunities to build capacity. | Young partners who were interested were trained when they were engaged in political activities (which was optional). The training focussed on conversation strategies. Young partners who were interested were also offered to follow a year-long program on advocacy.  Young partners could join a social media training as well as the opportunity to engage in and learn from doing research. | No formal training.  In some steps of the study, some explanation of aspects about the research project were provided. | Young partners were provided training in research skills and disability rights.  Throughout the project, young partners were encouraged to take on more leadership roles as and when they felt ready. Training was provided to support this e.g: presentation skills, media training, budgeting.  Throughout the project their lived experience and unique insight was fully recognised.  The project adopted an iterative approach so that we learnt together how we could fully ensure that this was co-led research by disabled young people. | Young partners were provided training in research skills and disability and children’s rights.  Throughout the project, young partners were encouraged to take on more leadership roles when they felt ready. Training was then tailored to meet needs, for example, presentation skills, media training, and/or budgeting.  Throughout the project, the lived experience and unique insight from young partners were fully recognised.  The project adopted an iterative approach so that we learn together how we continue to fully ensure that this project was a co-led research collective. | No formal training. One young partner and LN completed the Family Engagement in Research (FER) course offered by McMaster University, CanChild, and Kids Brain Health Network [3]. | No formal training. |
| **Proportional:** Involvement is tailored to the needs of the research and partners, pragmatic decisions are made to balance contradicting demands and limited resources.  Check-in meetings. | There were six physical panel meetings per year, which included informal breaks. Moreover, there were two fun activities each year and two calls for the position of the chair each year (2019).  Aside from the panel meetings, young partners were asked if they were interested to join projects, meetings or conferences. Young partners could contact the chair about their involvement, including changes, at any time. Based on who wanted to be involved in certain themes or activities, there were small group or individual communication, to discuss needs, roles, or time. With this approach, there were ‘theme groups’ and ‘commissions’ that began within the CFP Youth Panel. | The project group of researchers and BOSK coordinators decided on the needs of the research, and asked young partners who wanted to be involved in specific activities. The group communication tool was used to provide a short description of the next step and activities. Young partners decided if they were interested in specific activities. They could always contact the BOSK coordinator or researcher if they had any questions. Based on who wanted to be involved, there was small group or individual communication to discuss the needs of the research, as well as the role and time involved from each young partner. | Throughout the project, young partners were co-leaders. They decided together the level of involvement that each young partner wanted at each stage of the research cycle. This meant that individual skills and interests could be developed, and training provided at each stage. For example, some young partners wanted to undertake fieldwork, while others preferred to plan the launch of a conference. The face-to-face meetings ensured that all decisions and information were shared, and the project was owned by everyone. | Throughout the project, young partners were co-leaders and decided together on what work to undertake, and the ways in which the group were involved in individual projects. Then individually they decided on their own personal level of involvement across the whole research cycle. As a team, their collective skills and interests ensured that projects were delivered successfully.  The project operated within the social model of disability, which meant that any barriers they might face individually or collectively to being involved were addressed. | There were individual check-in meetings held biannually to discuss the goals and expectations of the SibYAC. The goals shared by individual SibYAC members were compiled and shared at a group meeting, to then discuss group goals. The check-in meetings were guided by two activities:   1. Start, Stop, Continue activity 2. Involvement Matrix [4] with the Patient Engagement Tool [5]. | There were individual check-in meetings held biannually to discuss the goals and expectations of all PFAC members, including young partners. The check-in meetings were guided by two activities:   1. Start, Stop, Continue activity, where feedback is provided about how we are doing as a PFAC as well as changes that would like to be implemented. 2. Involvement Matrix [4]. |
| **Communication:** Needs to be responsive and proactive.  Modes of communication. | Physical meetings, Email for monthly updates and when meetings approached, Whatsapp, and phone calls. | Email, Skype, WhatsApp, a platform called “Schriftje” where documents could be shared, and physical meetings | Physical meetings and phone/email communication between sessions. The communication style with young partners was tailored to the access needs of each young partner. | Physical meetings and phone/email communication between sessions. The communication style with young partners was tailored to the access needs of each young partner. | Email, Zoom, Facebook group, and synchronous meetings. | Email, Zoom, and Facebook messenger. |
| **What could have been done differently?** | Over time, the CFP Youth Panel shifted from being an advisory body to a leading body. Sometimes, it was also unclear whether the panel was an “advisor” or a “decider” on certain topics/activities of the CFP Program.  It would have also been good to have more (internal) evaluation on what this change meant, who did what for what reasons and how this worked in practice. Based on enthusiasm and time pressure, we often just “went for it”, without asking why and how. This sometimes led to conflicting expectations between the program and young partners.  Young partners were appreciated by the CFP program team, researchers, stakeholders, and project leaders. However, it is important to note that this change in attitude took some time (it was not always seen from the start on) and depended on context/project leader/etc. For example, in some situations only one panel member was asked to join a guidance committee for research and was therefore overshadowed by other ‘grown-up’ experts. | During the project, while reflecting on the partnership, young partners reported that the ‘how’ (how they could play a role) was not always clear, especially in the first stages, and in between meetings.  We learned how important it is to now and then together take a step back to reflect on the process, and on personal needs, wishes and ambitions.  Based on these personal ambitions some kind of training would have been appreciated by some of the ambassadors.  We also learned how helpful it can be to include young partners in the preparation of meetings too. | In both the VIPERS and RIP:STARS Projects, there could have been more young partners involved, which is dependent on funding.  The young people always want more opportunities for face-to-face meetings. although these were held monthly.  There could be more time to reflect on the process of involving young people in research and the contributions from young people.  In the RIP:STARS Project, subsequent recruitment will be via voluntary sector organisations and advocacy groups across England and through the RIP:STAR social media platforms. | | To ensure clear and transparent communication about roles and expectations, we could have created the documents on the Terms of Reference and Group Rules at the beginning of the study. Moving forward, it is important for us to keep track of demographics of our young partners (e.g., age, gender, race, ethnicity), as well as have a log of how young partners have been involved in different initiatives throughout the project.  While we were intentional in our recruitment to have young partners who were young adult siblings of individuals with a disability, we could consider recruiting more diverse young partners moving forward. For example, our current young partners include 5 sisters and 1 brother, and we could include more individuals from different genders. | It would have been great to have a meeting “buddy” system, so that new partners joining the project could be paired with a more experienced partner who could act as a mentor for onboarding. The mentor could also be a contact person to ask questions, provide resources, or provide feedback.  We could have also encouraged the use of different formats for sharing ideas, for example, online whiteboards such as Google Jamboards or typing in the chat.  While reflecting on the partnership to date, we had challenges at the beginning of the project about understanding patient-oriented research. There could have been more conversations about what patient-oriented research and co-design means to each partner, as well as how researchers and partners can support each other. It would be important to balance the time for reflection and training, while also meeting milestones of the project. It is important to recognize that we are learning on the go! |

**References**

1. Liabo K, Boddy K, Bortoli S, Irvine J, Boult H, Fredlund M, et al. Public involvement in health research: what does ‘good’ look like in practice? Res Involv Engagem. 2020;6:11.

2. CHILD-BRIGHT Network. Guidelines for Patient Partner Compensation [Internet]. 2020 [cited 2022 Apr 6]. Available from: https://www.child-bright.ca/compensation-guidelines

3. CanChild Centre for Childhood Disability Research. Family Engagement in Research Course [Internet]. 2022 [cited 2022 Feb 8]. Available from: https://www.canchild.ca/en/research-in-practice/family-engagement-in-research-course

4. Smits D-W, van Meeteren K, Klem M, Alsem M, Ketelaar M. Designing a tool to support patient and public involvement in research projects: the Involvement Matrix. Res Involv Engagem. 2020;6:30.

5. Ontario Brain Institute. Ways community members can participate in the stages of research. 2019.
