# Supplementary figures and images for: Exploring the “how” in research partnerships with young partners by experience: lessons learned in six projects from Canada, the Netherlands, and the United Kingdom
Source: Res Involv Engagem. 2022 Nov 17;8:62. doi: 10.1186/s40900-022-00400-7 (PMC9672637; doi:10.1186/s40900-022-00400-7)

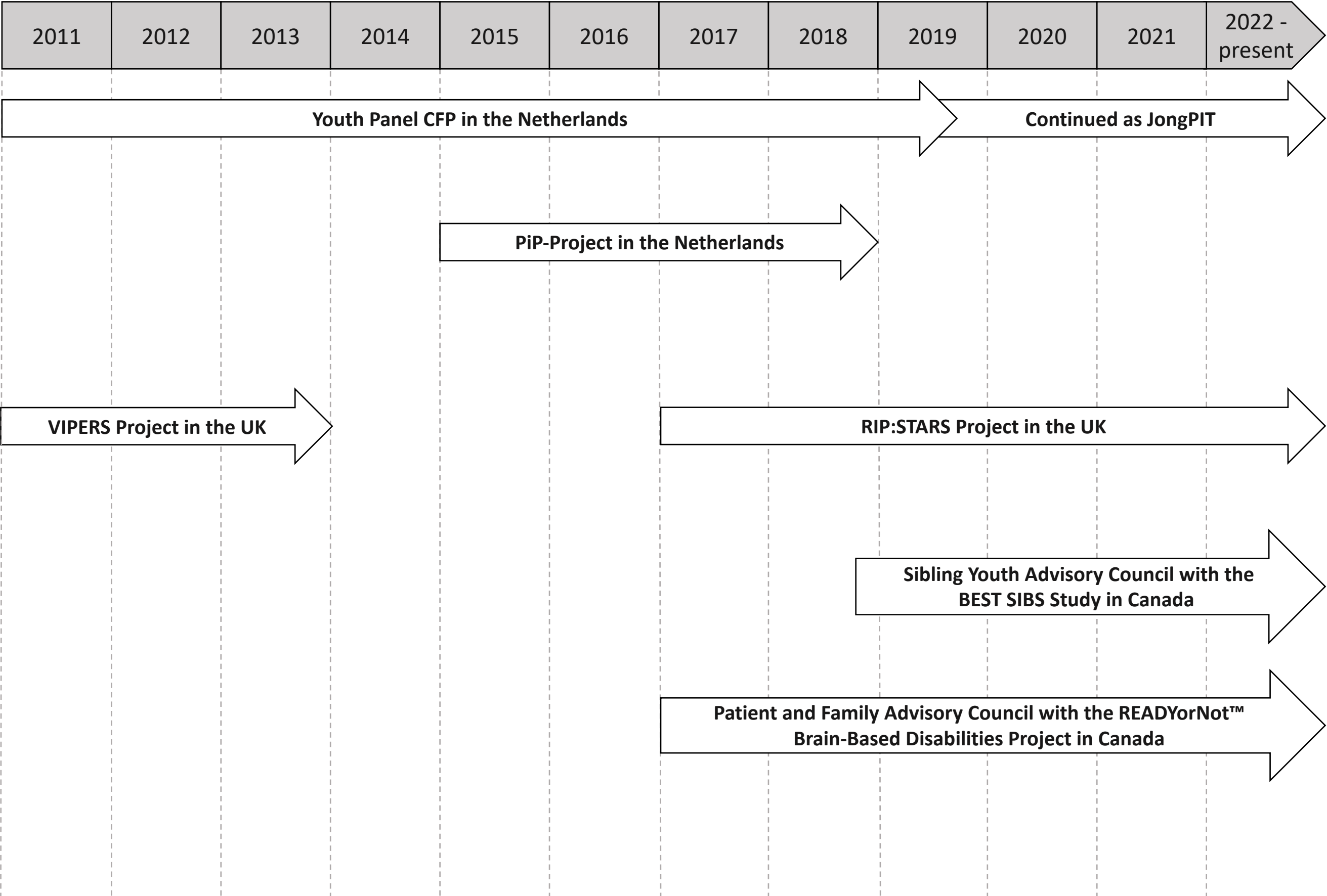

Supplement: Supplementary file 5 — Additional file 5. Length of time of involvement in projects. [file 40900_2022_400_MOESM5_ESM.pdf]
